# Modeling of *Zymomonas mobilis* central metabolism for novel metabolic engineering strategies

**DOI:** 10.3389/fmicb.2014.00042

**Published:** 2014-02-05

**Authors:** Uldis Kalnenieks, Agris Pentjuss, Reinis Rutkis, Egils Stalidzans, David A. Fell

**Affiliations:** ^1^Institute of Microbiology and Biotechnology, University of LatviaRiga, Latvia; ^2^Department of Computer Systems, Latvia University of AgricultureJelgava, Latvia; ^3^SIA TIBITJelgava, Latvia; ^4^Department of Biological and Medical Sciences, Oxford Brookes UniversityOxford, UK

**Keywords:** stoichiometric modeling, elementary flux modes, kinetic modeling, systems biology, metabolic engineering, Entner–Doudoroff pathway, central metabolism, *Zymomonas mobilis*

## Abstract

Mathematical modeling of metabolism is essential for rational metabolic engineering. The present work focuses on several types of modeling approach to quantitative understanding of central metabolic network and energetics in the bioethanol-producing bacterium *Zymomonas mobilis*. Combined use of Flux Balance, Elementary Flux Mode, and thermodynamic analysis of its central metabolism, together with dynamic modeling of the core catabolic pathways, can help to design novel substrate and product pathways by systematically analyzing the solution space for metabolic engineering, and yields insights into the function of metabolic network, hardly achievable without applying modeling tools.

## INTRODUCTION

*Zymomonas mobilis*, a member of the family of *Sphingomonadaceae*, is an unusual facultatively anaerobic Gram-negative bacterium, which has a very efficient homoethanol fermentation pathway. High ethanol yields, outstanding ethanol productivity (exceeding by 3–5 fold that of yeast; see Rogers et al., 1982), and tolerance to high ethanol and sugar concentrations, keep *Z. mobilis* in the focus of biotechnological research over four decades. The complete genome sequence of *Z. mobilis* ZM4, consisting of a single circular chromosome of 2,056,416 bp, was reported by Seo et al. (2005), followed by the genomes of several other strains (Kouvelis et al., 2009; Pappas et al., 2011; Desiniotis et al., 2012). Its small genome size, together with high specific rate of sugar catabolism via the Entner–Doudoroff (ED) pathway, and a relatively simple central metabolic network, make *Z. mobilis* a promising candidate for metabolic engineering (Sprenger, 1996; Rogers et al., 2007). Currently, recombinant *Z. mobilis* capable of fermenting pentose sugars is regarded as a potential alternative to yeast and recombinant *Escherichia coli* for ethanol biofuel synthesis from agricultural and forestry waste (Dien et al., 2003; Panesar et al., 2006; Rogers et al., 2007; Lau et al., 2010).

In spite of the seeming simplicity of its metabolism, *Z. mobilis* is a bacterium with an interesting physiology (Kalnenieks, 2006), posing researchers some long-standing challenges. Its extremely rapid glucose catabolism, far exceeding the biosynthetic demands of the cell, and the presence of an active respiratory chain with a low apparent P/O ratio (Bringer et al., 1984; Strohdeicher et al., 1990; Kalnenieks et al., 1993) are major manifestations of its so-called uncoupled growth. There are serious gaps in our understanding of the mechanistic basis of uncoupled growth, and in particular, the reason for the low degree of coupling in the respiratory chain of *Z. mobilis*.

**FIGURE 1:**
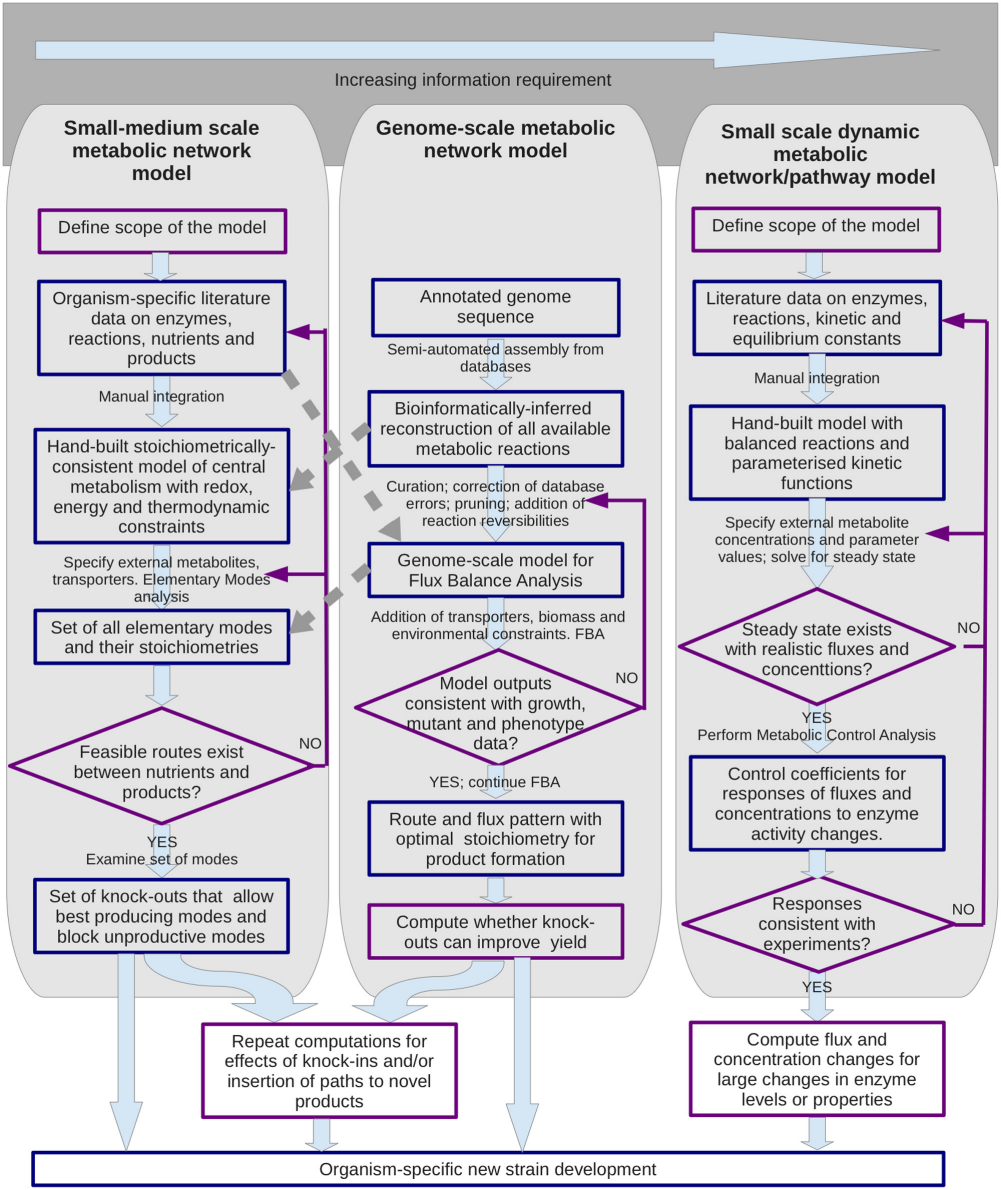
**Metabolic modeling support for metabolic engineering strategies.** Three main types of modeling approach are shown that have been used to analyze metabolic networks and to design alterations. The methodologies for the two structural modeling approaches shown on the left and center can have some cross-talk as indicated by the broken arrows. Blue boxes indicate entities such as data sets and models; purple outlines indicate procedures.

Mathematical modeling and *in silico* simulations are the most powerful tools of systems biology for understanding of complex metabolic phenomena, and often lead to novel, counterintuitive conclusions. A quantitative picture of physiology and metabolism is a key for rational, model-driven metabolic engineering. Some of the different metabolic modeling approaches that can support the design of novel metabolic engineering strategies are summarized in **Figure 1** (for reviews see: Schuster et al., 2000; Liu et al., 2010; Santos et al., 2011; Schellenberger et al., 2011; Rohwer, 2012). Compared with qualitative, pathway-oriented approaches, computational network analyses can enforce strict mass, energy and redox balancing and give an overall stoichiometric equation for predicted conversions (c.f. de Figueiredo et al., 2009). Here we outline recent advances and perspectives from applying such systems biology approaches to the physiology of *Z. mobilis*. We discuss some recent results gained by stoichiometric and kinetic modeling of its central metabolism, and their potential application to the design of novel substrate pathways, synthesis of novel products, and to the study of the uncoupled growth phenomenon *per se*.

## RECONSTRUCTION OF *Z. mobilis* CENTRAL METABOLIC NETWORK

Two medium-scale (Tsantili et al., 2007; Pentjuss et al., 2013) and two genome-scale stoichiometric reconstructions of *Z. mobilis* (Lee et al., 2010; Widiastuti et al., 2010) have been reported so far, representing instances of the left and center panels of **Figure 1** respectively. These reconstructions were based on the available genome annotation (Seo et al., 2005; Kouvelis et al., 2009) and provided an overall picture of *Z. mobilis* metabolism. The recent reconstruction made by Pentjuss et al. (2013) was focussed solely on the reactions of central metabolism and for the first time for *Z. mobilis* provided simulation-ready model files. That decreased the scale, yet allowed an improvement in the accuracy of reconstruction, by combining the genome-derived information with the preexisting biochemical evidence on *Z. mobilis*, available mostly for the reactions of catabolism and central metabolism.

Notably, several key reactions of central metabolism, common for the majority of the chemoheterotrophic, facultatively anaerobic bacteria, are absent in *Z. mobilis*. The Embden–Meyerhof–Parnas (EMP) glycolytic pathway is not operating in this bacterium. Absence of the EMP pathway has been confirmed by [1-^13^C]glucose experiments (Fuhrer et al., 2005), and furthermore, the gene for phosphofructokinase is lacking in the genome (Seo et al., 2005). *Z. mobilis* is the only known microorganism that uses the ED pathway anaerobically in place of the EMP glycolysis. Since the EMP pathway produces two ATP per glucose while the ED produces only one, it might seem that *Z. mobilis* suffers from ATP deficiency. However, it has been recently shown by means of thermodynamic analysis that, for a given glycolytic flux, the ED pathway requires significantly less enzymatic protein than the EMP pathway (Flamholz et al., 2013). On the other hand, the amount of the ED pathway enzymes in *Z. mobilis* cell is reported to be very high, reaching 50% of the cell’s soluble protein (Algar and Scopes, 1985; An et al., 1991). The high level of expression of the pathway together with its inherent speed, therefore, makes ATP production by the *Z. mobilis* ED pathway very rapid and, in fact, excessive for the needs of cell. Energy dissipation in order to regenerate ADP is thus essential for its balanced operation (Kalnenieks, 2006).

The TCA cycle is truncated, and consists of two branches, leading to 2-oxoglutarate and fumarate as the end products (Bringer-Meyer and Sahm, 1989). The genes for the 2-oxoglutarate dehydrogenase complex and malate dehydrogenase are absent (Seo et al., 2005), and accordingly, ^13^C-labeling patterns of 2-oxoglutarate and oxaloacetate do not support cyclic function of this pathway in *Z. mobilis* (de Graaf et al., 1999). Also, the pentose phosphate pathway is incomplete: transaldolase activity is lacking (Feldmann et al., 1992; de Graaf et al., 1999). The activity of 6-phosphogluconate dehydrogenase, the first reaction of the oxidative part of the pentose phosphate pathway, was reported to be very low (Feldmann et al., 1992). Subsequently, the corresponding gene (*gnd*) could not be identified in the sequenced genomes.

The aerobic redox cofactor balance and the function of electron transport chain represent yet another part of *Z. mobilis* metabolism that differs from that typically found in other bacteria. *Z. mobilis* is one of the few known bacteria in which both NADH and NADPH can serve as electron donors for the respiratory type II NADH dehydrogenase (ZMO1113; Bringer et al., 1984; Strohdeicher et al., 1990; Kalnenieks et al., 2008). Because of the truncated Krebs cycle, the ED pathway is the only source of reducing equivalents in catabolism, and therefore the electron transport chain competes for the limited NADH with the highly active alcohol dehydrogenases (Kalnenieks et al., 2006). Withdrawal of NADH from the alcohol dehydrogenase reaction would cause accumulation of acetaldehyde, which inhibits growth of aerobic *Z. mobilis* culture (Wecker and Zall, 1987). Nevertheless, this bacterium possesses a respiratory chain with high rates of oxygen consumption. The apparent P/O ratio of its respiratory chain is low (Bringer et al., 1984; Kalnenieks et al., 1993) though the mechanistic basis for that is not clear. However, for metabolic engineering purposes, an active, yet energetically inefficient electron transport has advantages for the needs of redox balancing during synthesis of novel products via metabolic pathways for which regeneration of NAD(P)^+^ is essential, whereas the aerobic increase of biomass yield is unwanted.

## QUEST FOR NOVEL SUBSTRATES AND PRODUCTS: STOICHIOMETRIC AND THERMODYNAMIC ANALYSIS

Much of the metabolic engineering in *Z. mobilis* has been devoted to broadening of its substrate spectrum and expanding its product range beyond bioethanol with a particular focus on the pathway of pentose sugar utilization for synthesis of bioethanol (Sprenger, 1996; Rogers et al., 2007). Advanced pentose-assimilating strains of *Z. mobilis* have been developed during the last couple of decades that can, in several respects, compete with the analogous recombinant strains of *E. coli* and *S. cerevisiae* (Lau et al., 2010). We were interested to explore the biotechnological potential of the low-efficiency respiratory chain of this bacterium for expanding its substrate and product spectrum.

Based on a medium-scale reconstruction of central metabolism (Pentjuss et al., 2013), stoichiometric modeling was used to search the whole solution space of the model, finding maximum product yields and the byproduct spectra with glucose, xylose, or glycerol as the carbon substrates for respiring cultures (**Figure 1**, left hand side). This was done by Flux Balance Analysis approach, using the COBRA Toolbox (Schellenberger et al., 2011). The stoichiometric analysis suggested several metabolic engineering strategies for obtaining products, such as glycerate, succinate, and glutamate that would use the electron transport chain to oxidize the excess NAD(P)H, generated during synthesis of these metabolites. Oxidation of the excess NAD(P)H would also be needed for synthesis of ethanol from glycerol.

It is essential, however, to complement the stoichiometric analysis with estimation of the thermodynamic feasibility of the underlying reactions. Glycerol utilization can serve as an example. Being a cheap, renewable carbon source, a byproduct of biodiesel technology, glycerol represents an attractive alternative substrate for *Z. mobilis* metabolic engineering. It is not expected to have serious growth-inhibitory effects, and also, little genetic engineering seems to be needed to make it consumable by *Z. mobilis*, and to channel it into the rapid ED pathway. Conversion of glycerol to ethanol by *Z. mobilis* would require expression of a heterologous transmembrane glycerol transporter and a glycerol kinase. Its genome contains genes for the two subsequent conversion steps, glycerolphosphate dehydrogenase and triose phosphate isomerase, leading to the ED intermediate glyceraldehyde-3-phosphate although, their overexpression might be needed. The further reactions from the glyceraldehyde-3-phosphate to ethanol represent a part of *Z. mobilis* natural ethanologenic pathway, and should be both rapid and redox-balanced. The extra NAD(P)H, generated by the glycerolphosphate dehydrogenase reaction could be oxidized by the respiratory chain. If succinate is the desired product (Pentjuss et al., 2013), the extra reducing equivalents could be used for reduction of fumarate by the respiratory fumarate reductase.

The pathway from glycerol to glyceraldehyde-3-phosphate via phosphorylation and following oxidation and isomerization steps is presented in biochemistry textbooks as the pathway of glycerol catabolism after breakdown of triacylglycerols in higher animals and humans (see e.g., Lehninger Principles of Biochemistry, 6th edition, Fig. 17-4). Though feasible from the stoichiometric point of view, it reveals problems when subjected to thermodynamic analysis. The equilibrium of the glycerolphosphate dehydrogenase reaction appears to be shifted very much toward formation of glycerol phosphate. *In silico* kinetic simulations of glycerol uptake for a putative engineered *Z. mobilis* demonstrate a dramatic accumulation of glycerol-3-phospate, reaching concentrations of several molar even at a high rate of NAD(P)H withdrawal by the respiratory chain (Rutkis et al., unpublished). Apparently, while the estimated overall stoichiometry of aerobic glycerol conversions is correct, thermodynamic analysis suggests the need to search for alternative reaction sequences to avoid excessive intracellular accumulation of metabolites.

## AEROBIC ELEMENTARY FLUX MODES OF THE PENTOSE PHOSPHATE PATHWAY

A metabolic network can function according to many different pathway options. Elementary flux mode (EFM) analysis has emerged as a systems biological tool that dissects a metabolic network into its basic building blocks, the EFMs (Schuster et al., 2000, 2002). All metabolic capabilities in steady states represent a weighted average of the EFMs, which are the minimal sets of enzymes that can each generate a valid steady state. The EFM approach has proved to be efficient for designing sets of knock-out mutations in order to minimize unwanted metabolic functionality in the producer strains. For example, in engineered *E. coli*, EMF-based mutation analysis helped to eliminate catabolite repression and to increase carbon flux toward the target product ethanol (Trinh et al., 2008).

**FIGURE 2:**
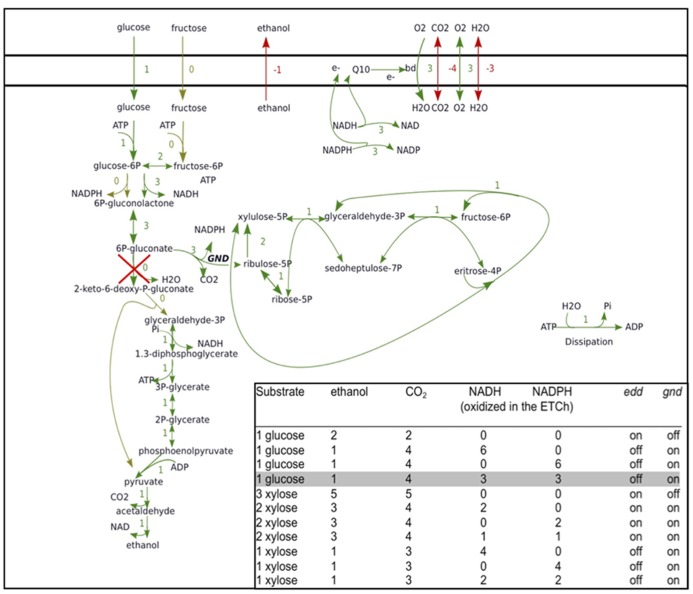
**Elementary flux modes of aerobic glucose and xylose catabolism for a strain with engineered pentose phosphate pathway enzymes.** Elementary flux mode for catabolism of glucose in cells with knocked-out *edd* and overexpressed heterologous *gnd* via the Entner–Doudoroff and pentose phosphate pathway, involving both NADH- and NADPH-oxidizing activity of the respiratory chain, is shown. ScrumPy modeling software EFM drawing algorithm (Pentjuss et al., unpublished) was used for visualization. Inset: complete list of elementary flux modes of glucose and xylose catabolism in *Z. mobilis*, involving the Entner–Doudoroff pathway, pentose phosphate pathway and the respiratory chain, with ethanol and carbon dioxide as the sole products. The explicitly shown elementary flux mode is shaded in gray.

By decomposing a network of highly interconnected reactions, the EFM analysis may reveal unexpected flux options. Recently we applied EFM analysis to the interaction between the ED, pentose phosphate pathway and respiratory chain in an engineered *Z. mobilis*, which expresses heterologous *gnd *and enzymes for pentose conversion, using the metabolic modeling package ScrumPy (Poolman, 2006). We were interested in the EFMs that such non-growing engineered *Z. mobilis* might employ for aerobic catabolism of glucose and xylose. Analysis revealed several EFMs in respiring cells (**Figure 2**) that have considerable interest for study of aerobic energy-coupling in this bacterium. With both monosaccharides, knocking out *edd* (encoding 6-phosphogluconate dehydratase), and overexpressing heterologous *gnd* (encoding 6-phosphogluconate dehydrogenase), would lead to generation of additional NAD(P)H and CO_2_ in the pentose phosphate pathway, while lowering the ethanol yield. Yet, most importantly, decrease of the ethanol yield would not be accompanied by accumulation of acetaldehyde and acetoin.

Thus, a simple EFM analysis suggests how to modify *Z. mobilis* aerobic metabolism so that its electron transport chain would receive more reducing equivalents without accumulation of inhibitory byproducts. Strains with such metabolic modifications might be very useful for study of the mechanisms underlying the uncoupled mode of oxidative phosphorylation in this bacterium.

## KINETIC MODELING OF THE ENTNER–DOUDOROFF PATHWAY

Despite the diverse studies of *Z. mobilis *physiology and genetics, little has been done so far to combine the accumulated knowledge in a form of kinetic model of central metabolism that would be comparable to the existing models for *E. coli* and yeast, and could be used to develop efficient metabolic engineering strategies (c.f. **Figure 1**, right-hand panel). A kinetic model reported by Altintas et al. (2006) focussed mainly on the interaction between the heterologous enzymes of pentose phosphate pathway and the native *Z. mobilis *ED glycolysis. Providing predictions for optimization of expression levels of the heterologous genes, this study contributed to strategies for maximizing xylose conversion to ethanol. However, the authors assumed constant intracellular concentrations of all adenylate cofactors. Since the ED pathway itself is a major player in ANP and NAD(P)(H) turnover, this might lead to erroneous conclusions on the pathway kinetics and restrict the range of model application. The recent kinetic model by Rutkis et al. (2013): (i) treated the cofactor levels as variables, making the interplay between adenylate cofactor levels and the pathway kinetics explicit, and (ii) introduced equilibrium constants in the kinetic equations to account for the reversibility of reactions more correctly. Metabolic control analysis (MCA) carried out with the model pointed to the ATP turnover as a major bottleneck, showing that the ATP consumption (dissipation) exerts a high level of control over glycolytic flux under various conditions (Rutkis et al., 2013).

Indeed, experimental studies of the ED pathway flux have shown that moderate overexpression of the ED pathway and alcohol dehydrogenase genes do not affect the glycolytic flux (Arfman et al., 1992; Snoep et al., 1995). Larger increases of the expression levels even caused a decrease in flux, exerting also a negative impact on *Z. mobilis *growth rate (Snoep et al., 1995). This clearly indicated that glycolytic flux in *Z. mobilis *must be controlled at some point(s) outside the ED pathway itself. The negative effects of overexpression apparently did not result from intrinsically negative flux control coefficients of the ED enzymes, but were attributable to the protein burden effect (Snoep et al., 1995), whereby overexpression of an enzyme with a small flux control coefficient caused reductions in the expression of other enzymes that have a greater influence on the flux. These results together with MCA studies on the kinetic model suggested that, due to the negligible flux control coefficients for the majority of reactions, single enzymes of the ED pathway should not be considered as prime targets for overexpression to increase the glycolytic flux in *Z. mobilis *(Rutkis et al., 2013). The calculated effects of several glycolytic enzyme (*gap, pgk, pgm*) and both alcohol dehydrogenase isoenzyme**(*adhA* and* adhB*) overexpression, in accordance with previous experimental observations, predicted little or no increase of glycolytic flux (Arfman et al., 1992; Snoep et al., 1995). The somewhat higher flux control coefficient for the pyruvate decarboxylase (*pdc*) reaction suggested that overexpression of this enzyme by more than 3-fold, might lead to an increase of glycolytic flux of almost 23% (Rutkis et al., 2013). However, quite the opposite was observed experimentally: approximately 10-fold increase of *pdc* was shown to slow down glycolysis by up to 25%, thereby implying that the protein burden might be a serious side effect of catabolic enzyme overexpression in *Z. mobilis*. Usually effects of protein burden are of minor importance in optimization of catabolic fluxes, due to relatively low concentrations of the enzymes in catabolic routes. This is not the case for *Z. mobilis* catabolism,**however, since**over 50% of the cell protein already is engaged in the function of the ED pathway (Algar and Scopes, 1985). Fortunately, flux control coefficient estimations still indicate a certain solution space for flux improvement: simultanous overexpression of *pdc, eno, pgm *within the 3-fold range of initial enzyme activities (wich most probably would be below the putative protein burden threshold), has the potential to increase the glycolytic flux by up to 25% (to reach 6.6 *g* glucose, *g* dry wt^-^^1^h^-^^1^; Rutkis et al. (2013).

Obviously, another option would be to raise ATP dissipation. That could be done by overexpression of the H^+^-dependent F_0_F_1_-ATPase, a major ATP-dissipating activity. Reyes and Scopes (1991) have estimated the F_0_F_1_-ATPase contribution being over 20% of the total intracellular ATP turnover. It should be noted, however, that overexpression of ATP-dissipating reaction(s) might disturb the intracellular ATP homeostasis, with successive suspension of glycolysis (by slowing down the first reaction of the ED pathway, phosphorylation of glucose). Co-response analysis indicates (Rutkis et al., 2013) that, at the highest glycolytic flux considered (4.6 g/g/h), the cellular capacity to maintain the ATP homeostasis is close to its limit, since even 1% further increase of glycolytic flux due to rise of ATP dissipation would be associated with a 4% decrease in ATP concentration.

## CONCLUSION

Although *Z. mobilis* metabolism has been subject to extensive research, and genome sequence data for several strains are now also available, it is only quite recently that modeling of its central metabolic network has started to gain momentum. These latest results of modeling *Z. mobilis* illustrate the relevance of combined stoichiometric, thermodynamic and kinetic analysis of central metabolism at different scales for microorganisms producing biorenewables. Concerted application of structural and dynamic modeling will help to identify targets for future metabolic engineering in a systematic manner, and provide novel insights into the biotechnological potential of this bacterium.

## AUTHOR CONTRIBUTIONS

All authors have equally contributed to the manuscript and have accepted the final version to be published.

## Conflict of Interest Statement

The authors declare that the research was conducted in the absence of any commercial or financial relationships that could be construed as a potential conflict of interest.
